# In-lab versus at-home activity recognition in ambulatory subjects with incomplete spinal cord injury

**DOI:** 10.1186/s12984-017-0222-5

**Published:** 2017-02-06

**Authors:** Mark V. Albert, Yohannes Azeze, Michael Courtois, Arun Jayaraman

**Affiliations:** 1 Max Nader Lab for Rehabilitation Technologies and Outcomes Research, Rehabilitation Institute of Chicago, Chicago, USA; 2 Department of Computer Science, Loyola University Chicago, Chicago, USA; 3 Department of Physical Medicine and Rehabilitation, Northwestern University, Evanston, USA; 4 Department of Biomedical Engineering, Illinois Institute of Technology, Chicago, USA

**Keywords:** Activity recognition, Activity tracking, Incomplete spinal cord injury, At-home, Machine learning

## Abstract

**Background:**

Although commercially available activity trackers can aid in tracking therapy and recovery of patients, most devices perform poorly for patients with irregular movement patterns. Standard machine learning techniques can be applied on recorded accelerometer signals in order to classify the activities of ambulatory subjects with incomplete spinal cord injury in a way that is specific to this population and the location of the recording—at home or in the clinic.

**Methods:**

Subjects were instructed to perform a standardized set of movements while wearing a waist-worn accelerometer in the clinic and at-home. Activities included lying, sitting, standing, walking, wheeling, and stair climbing. Multiple classifiers and validation methods were used to quantify the ability of the machine learning techniques to distinguish the activities recorded in-lab or at-home.

**Results:**

In the lab, classifiers trained and tested using within-subject cross-validation provided an accuracy of 91.6%. When the classifier was trained on data collected in the lab but tested on at home data, the accuracy fell to 54.6% indicating distinct movement patterns between locations. However, the accuracy of the at-home classifications, when training the classifier with at-home data, improved to 85.9%.

**Conclusion:**

Individuals with unique movement patterns can benefit from using tailored activity recognition algorithms easily implemented using modern machine learning methods on collected movement data.

## Background

Activity tracking can be performed using wearable sensors, which provide a wealth of information to encourage beneficial movement. Commercial activity tracking has gained immense popularity with a number of consumer devices available to help the general population track their fitness goals [1, 2]. Patients with motor disabilities, such as those with spinal cord injury, can benefit from activity tracking especially in therapeutic or clinical environments [3, 4]. Many of these devices, however, are not designed to work effectively for movement-impaired populations as they have not been validated in these populations. Precise and automatic activity recognition has the potential to help create and evaluate individualized treatments plans, but more work must be done to improve the experience for movement-impaired patient populations.

For individuals with spinal cord injuries, half of motor recovery occurs within the first few months and full neurological recovery occurs within 2 years of the injury [5]. In this time of recovery, the vast majority of individuals return home and are not institutionalized during recovery with only periodic assessments performed in a clinical setting [6, 7]. During the extended recovery phase, it can be beneficial to have accurate feedback on the quality and quantity of patient movements [6]. However, when patients return home, there is currently no standard practice of assessing the quality of their mobility without return clinical visits. Activity tracking offers individuals with incomplete spinal cord injury needed data on their movement patterns to improve therapies and monitor recovery.

There are obstacles, however, to bringing activity tracking to populations with motor disabilities. Consumer-oriented activity trackers typically perform limited analyses including calorie estimates, step count estimates, or general activity levels [1, 2]. These same activity trackers ignore physical symptoms of individuals with motor disabilities that include muscle spasticity and tremor. For clinical purposes, many consumer-oriented activity trackers currently do not monitor movements necessary to the therapies of these individuals that include lying, sitting, standing, wheeling and stair-climbing. Although these activity trackers are inexpensive, their limited, proprietary analyses are often not validated for use in a clinical setting for specific patient populations [7]. The direct analysis of 3-axis accelerometers allows for cheap, controlled, repeatable, and reliable activity recognition [8], but most commercial systems for movement analysis are designed to track the movements of healthy individuals. Due to the irregular movement patterns of individuals with motor disabilities, the probability of a misclassification is much higher. Alternative approaches to activity tracking are necessary to accurately and automatically analyze movements in such populations.

The current methods of evaluating patients with motor impairments have limits that motion tracking can address. Performing periodic clinical assessments requires daily, weekly, or monthly visits. These visits are time consuming for the subject and expensive both financially and strategically in terms of clinician time and effort. Journaling is another popular method for clinical evaluation. However, the inconvenience of periodic journaling results in a low compliance rate [9] and the subjective nature of journaling produces data which is less reliable than objective measures. Wearable motion tracking, by contrast, can be used inexpensively and unobtrusively at home and provides objective measures of performance.

Performing activity recognition in populations with motor disabilities is particularly challenging. However, activity recognition strategies can be tailored specifically for populations with unique movement patterns. For example, Parkinson’s disease symptoms including tremor, slowed motion, rigid muscles, loss of common automatic movements, and impaired posture [9] and such impaired movements have been shown to dramatically affect activity recognition algorithms [4]. Activity recognition has been performed for many populations, including the elderly [10], individuals with muscular dystrophy [11], and Parkinson’s disease [12, 13]. The studies demonstrate that it is possible to adapt activity recognition algorithms to unique patient populations.

An additional complication in using activity recognition is the discrepancy between movements recorded in the clinic under supervision when compared to at-home movements. When asked to complete a series of movements in a controlled clinical setting, patients will often move in a stereotypical way that is often dissimilar to their normal movement patterns. Much like the tailoring of activity recognition methods to impaired movement populations versus those with healthy mobility, activity recognition algorithms should also be tailored to the unique at-home movement profiles of subjects. This is especially important as accurate at-home data is more beneficial than accurate identification in clinical settings. Machine learning approaches that can be used to tailor activity recognition for unique patient populations can also be adapted to at-home movements with the right data available.

Here we tailor the activity recognition algorithms for a waist-worn accelerometer to the movements of patients with incomplete spinal cord injury. We recorded instructed movements both in the clinic and at home. Using this data, we compared and contrasted the accuracy of methods based on laboratory training alone versus an approach which included at-home data for training as well. By tailoring the activity recognition algorithms to not only the individual population, but also the at-home movements of that population, we expect much greater accuracy in tracking their movements in a context much more relevant to their everyday lives.

## Methods

Thirteen ambulatory subjects with incomplete spinal cord injuries (9 M/4 F, ages 22–50) participated in this study. Informed, written consent was acquired for all subjects. The Northwestern University Institutional Review Board approved this study.

**Fig. 1:**
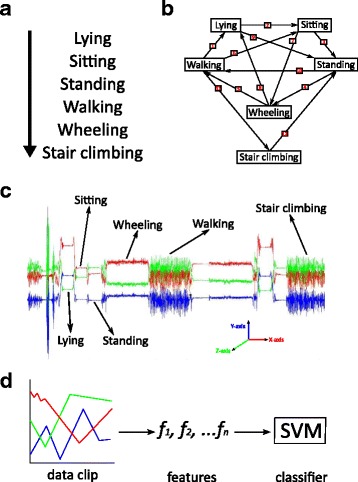
*Experimental protocol*. **a** At home, subjects performed the following set of physical activities in the order shown. **b** In the lab, subjects performed the physical activities in the displayed sequence in order to record every pair of transitions between activities. **c** Data from a tri-axial accelerometer was collected while performing these activities. **d** *Data processing*. A series of features were extracted from 10 s clips of data, and supervised machine learning was used to train an activity recognition classifier

Two instructed activity sets were developed for the subjects to carry out in-lab and at-home. In-lab data was collected while subjects were instructed to perform the following activities: lying, sitting, walking, standing, wheeling, and stair climbing. The order of these instructed activities, as shown in Fig. 1, was to allow every combination of transitions between activities. In order to capture movements at home, subjects were instructed to perform a similar set of activities in order, at three distinct times per day and record the time each activity set was performed. All subjects were instructed to wear an Actigraph wGT3X tri-axial accelerometer on their waist using a provided waist strap; the accelerometer sampled at a rate of 100 Hz with a dynamic range of +/− 8 g’s.

### Data processing and classification

The recorded accelerometer data was first labeled using an in-house developed MATLAB GUI. Activities were visually identified based on the accelerations and the expected temporal order of the instructed activities. The recordings were then parsed into ten second clips. One-thousand and One-hundred seventy samples were recorded in-lab and 1089 samples were recorded at-home. On these clips a standard set of time series features were automatically selected, weighed, and combined using standard machine learning classifiers, as summarized in Table 1.

**Table 1:** Features used for activity recognition

Description	Total number of values
Mean, absolute value of the mean	6
Moments: standard deviation, skew, kurtosis	9
Deviation, skew, kurtosis	12
Root mean square	3
Smoothed root mean square (5 pt kernel, 10 pt kernel)	6
Extremes: min, max, abs min, abs max	12
Histogram: includes counts for −4 to 4 z-score bins	27
Fourier components: 32 samples for each axis	96
Overall mean acceleration	1
Cross product means: xy, xz, yz	3
Abs mean of the cross products	3

Three validation methods were used to explore the influence of context on classification accuracy - cross-validation analysis of in-lab collected data, training on in-lab and testing on at-home data, and cross-validation analysis of at-home data. All three validation methods used 10-fold cross-validation over the entire set of subjects.

Six different classifiers were initially considered for classification: support vector machine; naïve bayes; regularized logistic regression; K nearest neighbors; and decision trees. The hyperparameters for the classifiers were found by a grid search of 10^×^ where × is an integer between −5 and 5 or using a suitable range specified below. Hyperparameters were selected by minimizing the 10-fold cross-validation using only in-lab activity but were robust to other validation methods. We applied the following the following parameters for the classifiers. The support vector machine (SVM) classifier yielded the most promising results. For SVM, we normalized each feature to have 0 mean and unit variance. We applied radial basis functions, giving us two hyperparameters—the soft slack variable, C, and the size of the Gaussian kernel, γ. The values found by cross validation were C = 10 and γ = 1. Naïve Bayes had no hyperparameters. K nearest neighbor employed a 1–30 search with a *k* value equal to 5. For the decision tree classifier, the minimum number of samples needed to split a node was found to be 10 after a 1–30 search. For logistic regression we used an L1 penalty (lasso) and a regularization strength, λ, of 0.01.

## Results

Our first goal was to predict the activities of individuals with incomplete spinal cord injury in a laboratory setting. Using in-lab activity data, we were able to accurately predict the activity with 91.6% accuracy (89.9–93.2%, binomial 95% confidence interval) (Table 2). Walking was the most accurately predicted with 97.0% recall followed by lying at 95.9% and wheeling at 95.8%. In-lab activity possessed an overall precision of 91.34% and a recall of 90%. The lower accuracies were the result of misclassification of similar physical activities. The recorded signals from walking and stair climbing are quite similar, with misclassification of each due to the other similar activity in 80.8% of the misclassifications. A similar pattern is seen for sitting and moving in the wheelchair (wheeling).

**Table 2:** Classification matrix for in-lab activity using the SVM classifier

Activity	Lie	Stand	Sit	Wheel	Walk	Stairs
Lie	**191**	5	2	0	1	2
Stand	5	**148**	8	0	1	1
Sit	5	8	**135**	14	0	0
Wheel	0	0	9	**239**	0	1
Walk	0	0	0	0	**259**	8
Stairs	0	2	0	0	28	**100**

Rows correspond to true activities, columns are predicted activities. Overall accuracy 91.6% (89.9–93.2%). The highest accuracy classifier for each validation method is indicated in bold

After assessing in-lab activity recognition, we assessed classification accuracy for data collected in the home. When using classifiers trained on the previous in-lab collected data, we would expect at-home activity recognition to be much lower due to the lack of supervision and increased variability in at-home movement patterns. Using at-home activity data for testing, accuracy was 54.6% (51.6–57.6%) (Table 3). Note, by combining the data of two easily misclassified similar activities, walking and stair climbing, accuracy increases roughly ten percent to 63.5%. However, that low percentage is based on at-home activity classification using in-lab collected data. If the classifier is trained using at-home data, the accuracy significantly improves to 85.9% (83.6–87.9%) using within-subject cross-validation and the SVM classifier.

**Table 3:** Classification matrix for in-lab training and at-home testing using the SVM classifier

Activity	Lie	Stand	Sit	Wheel	Walk	Stairs
Lie	**209**	60	39	28	6	8
Stand	1	**77**	29	6	1	5
Sit	27	22	**24**	11	0	3
Wheel	36	30	9	**39**	0	14
Walk	3	6	3	11	**134**	38
Stairs	0	8	0	8	72	**112**

Note the overall accuracy of 54.6% (51.6–57.6%) - a substantial reduction from in-lab only validation. The highest accuracy classifier for each validation method is indicated in bold

We tested the accuracy of multiple machine learning classifiers. Overall, the SVM classifier was most accurate with 91.6% for in-lab activity and 85.5% for at-home activity using within-subject 10-fold cross-validation (Table 4). Naïve Bayes performed with the highest accuracy at 91.8% for the in-lab only condition, although significantly dropping in accuracy in other categories (Table 5). Regularized logistic regression, K-nearest neighbor, and decision trees were also tested, but performed poorly.

**Table 4:** Classification matrix for at-home activity using the SVM classifier

Activity	Lie	Stand	Sit	Wheel	Walk	Stairs
Lie	**327**	5	4	5	5	4
Stand	7	**99**	6	2	1	4
Sit	12	10	**66**	6	2	1
Wheel	5	0	3	**116**	4	0
Walk	1	0	3	0	**161**	30
Stairs	3	1	0	0	30	**166**

Overall accuracy 85.9% (83.6–87.9%)

**Table 5:** Classification accuracy of each validation method shown in Tables 2, 3 and 4 using different classifiers

Validation method	SVM	Naive bayes	Logisitic regression	kNN	Decision tree
Within-subject, in-lab activity	91.2%	**91.9%**	90.8%	86.1%	89.6%
Within-subject, train in-lab, test at-home	**54.6%**	45.5%	47.7%	54.2%	49.0%
Within-subject, at-home activity	**85.6%**	79.4%	85.1%	79.6%	82.1%

The highest accuracy classifier for each validation method is indicated in bold

## Discussion

We recorded individuals performing a series of physical activities both in-lab and at-home and identified those activities using machine learning classifiers. Our results noted a large difference in recognition accuracy depending on the subject being in the lab or at home. We found that activities could be classified with 91.6% accuracy using within-subject cross-validation on data collected in the lab setting. The poorest classification accuracy was found when testing was performed at home using classifiers built from in-lab training data. To improve performance of at-home activity classification we demonstrated how at-home training data can be used to restore the accuracy.

There has been a significant body of research on the use of accelerometers in activity recognition for specific patient populations [14, 15], including the use of accelerometer-enabled smart phones which make activity tracking possible with only the download of an application on the phone [15–17]. Accelerometer-enabled activity recognition has been used in populations recovering from stroke [18]. The study participants wore triaxial accelerometers on their ankles in which machine-learning was used to recognize differences between walking, exercise, and cycling and speed. A similar study on stroke patients used an accelerometer placed in the shoe to automatically classify standing, sitting, and walking [19]. Activity recognition of this kind has also been used to monitor falls in elderly populations through accelerometers in mobile phones [20]. Mobile phone activity recognition was also successful applied in populations with Parkinson’s disease [4]. These studies have demonstrated that by using accelerometers on dedicated devices and smart phones it is possible to monitor activities in specific patient populations.

Our previous work using mobile phone activity recognition in Parkinson’s disease demonstrated the need to use population-specific activity classification [13]. Phone-based activity recognition for control subjects had an accuracy of 96.1% for a similar set of activities. When Parkinson’s patient movements were analyzed using the algorithm developed from the control subjects the accuracy was only 60.3%. However, when PD patient movements were used to train the classifier, accuracy was restored to 92.2%. Such a significant drop in accuracy, then subsequent improvement, reflects the necessity for patient population specific data analysis. Analogously, movements differ between the in-clinic and at-home environments, which suggested to us the need to collect data in these specific environments as well.

Similar to the Albert et al. 2012 study, the obtained classification accuracies suggest a need to collect movement data specific to the population and context. The highest accuracy for our incomplete SCI patients (91.6% within-subject) was obtained on the movement data collected in the laboratory setting. This is expected, as these movements were instructed, directly observed, and likely more stereotypical. This was also observed anecdotally in the variability in acquired accelerometer signals between in-lab and at-home samples. Training on movements collected in the lab and later testing on movements collected at home provided the least accurate classification (54.6% within-subject). Unfortunately, this reflects the potential inaccuracy of activity recognition systems designed and testing in a clinical setting. However, by including movement data measured at home in the training set, the accuracy was increased to 85.9% within-subject demonstrating an advantage to collecting at-home data for activity recognition.

The conclusions of this study are limited by aspects of data labeling and the large variations in patient disability. For consistency, labeling was performed by a single researcher; however this labeling was not evaluated for test-retest reliability, which would produce an upper bound on the accuracy of any classifier. Although all activities transitions were represented during in-lab recording, the at-home recordings were a fixed sequence for all participants; this limits generalizability, as there may be sequence-specific alterations to movement patterns. Video recordings, as opposed to labeling of accelerometer readings from fixed activity sequences, would improve the reliability of the labeling used for validation. As indicated in the classification matrices, some pairs of activities are more likely to be misclassified than others. Specifically, standing and sitting—as they are both sedentary and in the same orientation for many people—are more likely to be misclassified. A similar ambiguity exists between walking and stair climbing, as both classes have significant, similar movements. By labeling these classes more generally (e.g. active or sedentary) higher accuracies would be expected. Beyond the labeling concerns, subjects possessed a wide range of movement impairments—some used walkers while others required other forms of support. For wheelchair recognition, some subjects used joystick-controlled wheelchairs, which made wheeling motions almost indistinguishable from sitting in many of the clips. Although including these various levels of patient movement ability lead to lower accuracy, this set of data provides a better validation set due to its relationship to movements expected in practice.

## Conclusion

There were two main goals for this study. First, we demonstrate how activity recognition can easily be adapted to a population with a particular class of movement patterns, such as patients with incomplete spinal cord injury. Our second goal was to demonstrate that recording movements in the correct context is necessary for accurate activity recognition, especially in populations with limited mobility. Both population-specific models and context-trained models can be designed with specialized data sets to increase accuracy—by using modern machine leaning methods both population and context-specific models can be created using only a change in the data set. The improved ability to track patient activities can lead to better data-driven therapeutic interventions for better functional gains in mobility impaired individuals.

## Abbreviations

iSCI: Incomplete spinal cord injury
SVM: Support vector machine (classifier)
